# Supplementary material for: BIGMAC : breaking inaccurate genomes and merging assembled contigs for long read metagenomic assembly
Source: BMC Bioinformatics. 2016 Oct 28;17:435. doi: 10.1186/s12859-016-1288-y (PMC5084376; doi:10.1186/s12859-016-1288-y)
Supplement: Supplementary file 1 — Supplementary materials include implementation details, data analysis, theoretical analysis and commands used to run different tools. (PDF 492 kb) [file 12859_2016_1288_MOESM1_ESM.pdf]

Appendices

This appendix includes the following sections.

1 Implementation details of the break point finding algorithm
2 Data analysis of the Breaker and Merger
3 Feasbility of Breaker to recover consistent contigs
4 More information on the EM algorithm and the MSA
5 Commands used to run various tools
6 Detailed Quast reports
7 Future work

Appendix A: Implementation details of the break point finding algorithm

In forming a De Bruijn graph, we use the following method. First, we fill in the hidden end points by inspecting any inconsistent number of end points between repeat copies. In our example of $x_1 = a[b(c]d)e, x_2 = f[bc]g, x_3 = h(cd)i$, we have [()] as the long($\geq 2L$) repeat end points. We fill in the hidden end points $x_1 = a[b(c]d)e, x_2 = f[b|c]g, x_3 = h(c|d)i$ because between [] there should be a), and between (), there should be a]After filling in the hidden end points, we label and cluster the end points. At first, two end points have the same label if they correspond to the same side of the same repeat. Then, we cluster end points that are close to each other to have the same label. With the relabelled end points along each contig, we form a graph. Note that the end points correspond to edges of the graph. In the previous example, let the label of end point of [(]) be $1, 2, 3, 4$ respectively, we have the edge sequences of x_1, x_2, x_3 being $(1, 2, 3, 4), (1, 2, 3), (2, 3, 4)$. And we will append beginning and ending edge to the sequences, so the actual edge sequences of x_1, x_2, x_3 are $(b_1, 1, 2, 3, 4, e_1), (b_2, 1, 2, 3, e_2), (b_3, 2, 3, 4, e_3)$. Next, we need to find the nodes. This can be done by scannig for successive end points in the edge sequences. Any two successive end points define a node. And if they do not correspond to a closed end point followed by an open end point, it is considered as a repeat node. For example, $(1, 2)$ is a repeat node node, and $(b_1, 1)$ is a non-repeat node. Now we note that from the repeat nodes, we can gather together the edges to form the graph. For example, the incoming edges of node $(1, 2)$ are the two end points corresponding to 1 and outgoing edges of the node $(1, 2)$ are the two end points corresponding to 2. In order to handle double stranded nature of the genome, when scanning the edge sequences, we search both forward and backward to identify the nodes. The approximate nature of matching is handled when we cluster end points close to each other.

Appendix B: Data analysis of the Breaker and Merger

We perform independent data analysis of the performance of Breaker and Merger of BIGMAC. We note that we both use QUAST and an independent evaluation(which is implemented by us) from QUAST. Users can use our evaluation scripts to evaluate the performance of their own improvement as well. We note that the dataset 1,2,3 are those studied in the experiment section and the dataset 0 is the synthetic dataset.

B.1 Quast reports

The Breaker only and BIGMAC end-to-end results are tabulated as follows. We note that Breaker can decrease the number of contigs because it remove redundant contigs after breaking at potentially mis-assembled points. The are located at the QUAST report section.

B.2 Data anaysis on Breaker

We measure mis-assemblies fixing capability of Breaker. Specifically, we study the performance of ChimericContigFixing(Palindrome) and the combination of LocatePotentialMisassemblies and ConfirmBreakPoints (Repeat&Coverage). We map the contigs back to the ground truth to see if the segments mapped to different locations. We note that our method is more stringent that QUAST. Even in the cases of repeat, we only map the segment to the best matched location. Thus, occasionally, a FP may not be a real false positive. The script can be run as `python -m srcRefactor.evalmfixer foldername mummerpath` The precision and recall on the subcompoents are as follows.

Table 4: Breaker Evaluation

Dataset	Break point detector	Precision	Recall	Number of TP	Number of FP
0	Palindrome	1	0	0	0
0	Repeat&Coverage	1	1	2	0
1	Palindrome	1	0	0	0
1	Repeat&Coverage	0.102041	0.483871	15	132
2	Palindrome	0.605556	0.246606	109	71
2	Repeat&Coverage	0.021898	0.032967	9	402
3	Palindrome	0.818182	0.157895	9	2
3	Repeat&Coverage	0.142857	0.113636	5	30

B.3 Data anaysis on Merger

To evaluation, we collect data from graphsurgery merges(when condensing edges), BRepeat merges(when repeat node is not a separte node) and XRepeat merges(when repeat node is a separate node). We map back to reference to identify correct successors. Then we report the percentage left. The scripts can be run as `python -m srcRefactor.evalasplitter foldername mummerpath` The precision and recall on the subcompoents are as follows. Note that we are more stringent than QUAST, because if two are not immediate successors then we report as FP here. Also, we use best match on the reference, meaning that repeat can be mapped to more than one location, thus a FP may not really be a FP. So, the number reported only serves as an approximation here. We note that we have duplicated tje contigs to handle reverse complements, so all numbers are approximately double of the actual number, with some offset due to slight variation due to tie-breaking in the alignment tool.

Appendix C: Feasbility of Breaker to recover consistent contigs

In this section, we study why Breaker can recover contigs by modelling the mis-assemblies formed by an upstream assembler

We define the ground truth to be $S_0 = \{s_1, s_2, ..., s_n\}$ which is a set of strings with alphabets taken from $\Sigma = \{A, C, G, T\}$. Now we specify their repeat structures as follows. Let x, y be length L substrings of s_i, s_j respectively , where $i \neq j$ and

Table 5: Merger Evaluation

Dataset	Merger subroutine	precision	recall	TP_num	FP_num
0	GraphSurgery	1	0	0	0
0	BResolve	1	1	4	0
0	XResolve	1	0	0	0
1	GraphSurgery	0.829268	0.164251	68	14
1	BResolve	0.745455	0.099034	41	14
1	XResolve	0.823529	0.033816	14	3
2	GraphSurgery	0.741379	0.076512	43	15
2	BResolve	0.384615	0.008897	5	8
2	XResolve	0.250000	0.001779	1	3
3	GraphSurgery	0.235294	0.090909	4	13
3	BResolve	0.333333	0.045455	2	4
3	XResolve	1.000000	0.022727	1	0

$L > 2$. If $\forall 1 < k < L, x[k] = y[k]$ and $x[1] \neq y[1], x[L] \neq y[L]$, then we call (x, y) be a maximal exact repeat of length $L - 2$. Although this notion of maximal exact repeat can be generalized to the same string, for simplicity of discussion, we assume they are extracted from different strings. We fix K_0 to be a large constant which is related to the length of the reads and assume that there are only r maximal exact repeats of length $> K_0$.

Next, we model the upstream assembler's mis-assembly formation process by the following sequence of operations of strings. Let $\{T_j\}_{1 \leq j \leq m}$ be a sequence of operations that act on strings S_0 and form $\{S^{(j)}\}_{1 \leq j \leq m}$ successively. That is, $S^{(0)} = S_0$ and $1 \leq j \leq m, S^{(j)} = T_j(S^{(j-1)})$. Now, we specify the action of T_j. It picks two arbitrary strings with a maximal repeat of length ¿ K_0. Then, it breaks at the start of the repeat and joins the corresponding string at the breakpoint. Symbolically, let T operate on two strings $s = axb, t = cxd$, where the common segment is x and the breakpoint is the position immediately before x. The resultant strings are $s' = axd, t' = cxb$. We further assume that each string under the operations does not have repeat within itself of length $> K_0$.

Under this setting, we prove the following theorem.

Theorem C.1 *Given $S^{(m)}$ generated from $S_0 = \{s_i\}_{1 \leq i \leq n}$ after successive operations by $\{T_j\}_{1 \leq j \leq m}$, we can recover a set of strings W of cardinality at most $n + 4r$ such that W is consistent with S_0 (i.e. for each string $w \in W$, w is a substring of some string $s \in S_0$).*

Proof The way to construct the set W is as follows. We first identify all maximal exact repeats across the strings in $S^{(m)}$. We then break the strings at every endpoints of each of these maximal exact repeats. Now, it remains to show that 1) there are at most $n + 4r$ strings in W and 2) they are consistent with the ground truth.

To show them, we use the following bookkeeping method. Let us assign a unique label to each position at each string in the ground truth S_0. Let the set of all the labels be B and the mapping from B to string index and offset be f_0. At the beginning, we define Φ_0 as the labels that are the endpoints of any maximal exact repeat of length $> K_0$. That is, $\Phi_0 = \{a \in B \mid a \text{ corresponds to an endpoint of some maximal exact repeat of of length } > K_0 \text{ in } S^{(0)}\}$. When we apply T_j on the strings, let x be the repeat. We move

both the segment and the associated labels to the other string starting at the left endpoint of x. The exceptions are the labels within the repeat x which are associated with some right endpoints of another repeat x' that has left endpoint before x. We keep those labels at the original positions. Since the set of labels remains invariant, and they correspond to a bijection, f_j, from B to string position at each stage after T_j, we can define $\Phi_j = \{a \in B \mid a$ corresponds to an endpoint of some maximal exact repeat of of length $> K_0$ in $S^{(j)}\}$

We consider the simple case when initially no two pairs of repeat copies overlap at exactly one point(otherwise, we just need to generalize our book keeping scheme by introducing multiple labels at those points). In that case, it turns out that Φ_j is invariant(i.e. $\Phi_j = \Phi_0$ for all j), which we will prove in a separate Lemma. With this Lemma, then we can show the theorem follows.

We first show that W is consistent with S_0. We note that for each T_j, if we mark the label of the junction as b_j and break them, then the resulting set of string will be consistent throughout. But since $b_j \in \Phi_j$ and $\bigcup_j \{b_j\} \subset \bigcup_j \Phi_j = \Phi_m = \Phi_0$, it suffices to break at every position corresponding to Φ_m in $S^{(m)}$ to obtain consistent strings. Moreover, $|\Phi_m| = |\Phi_0| \leq 4r$. So, if we break at every position corresponding to Φ_m in $S^{(m)}$, we have at most $n + 4r$ resultant strings. This gives, $|W| \leq n + 4r$. □

Lemma C.2 *If* $0 \leq j \leq m$*, we have* $\Phi_j = \Phi_0$.

Proof We consider $j = 1$ and inductively, the lemma follows. Without loss of generality, we assume s_1, s_2 are the strings that T_1 acts on and the associated repeat is x.

If T_1 can cause an element $b \in B$ to enter or leave Φ_1, it could only belong to a maximal repeat that includes a copy of x. Otherwise the labels and the moving segment, which include that potential repeat segment, are moved together. Thus, there cannot be any creation/destruction of maximal exact repeats. We will show that, even for those repeats that include a copy of x, their endpoints are still invariant. Without loss of generality, we take the suspicious repeat to end at the right endpoint of s_1. There are two cases that can cause changes in Φ_1 upon T_1. These include getting a bigger maximal repeat or getting a big repeat separated into smaller pieces with a third string. Since we assume that we cannot have a repeat of length ¿ K_0 on the same string in the sequence of operations, the third string cannot be s_1 or s_2. They correspond to a T_1 that goes either from left to right or right to left in Fig 4. We enumerate the pairwise maximal repeats as shown in Fig 4. It turns out that in both cases, the set of associated repeat endpoints is invariant. This concludes the proof that $\Phi_1 = \Phi_0$ □

Appendix D: More information on the EM algorithm and the MSA

In this section, we discuss about the details of the EM algorithm used and related materials.

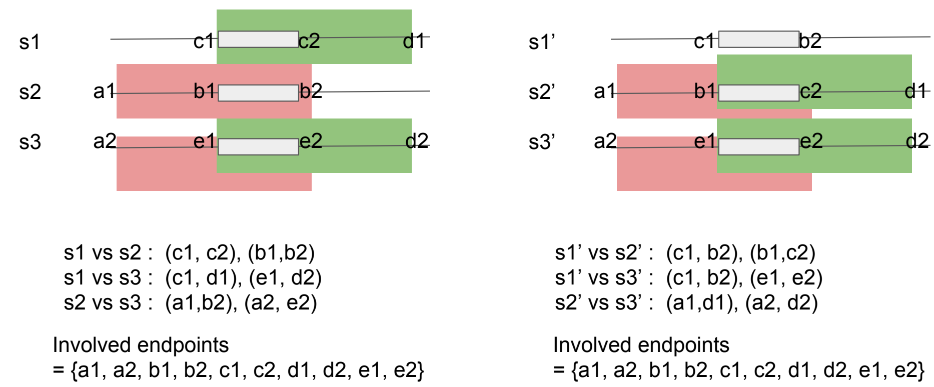

Figure 4: Illustration of conservation of endpoints

D.1 Derivation of the EM algorithm

$$
\begin{aligned}
&\log P_\theta(X, Z) \\
&= \log \Pi_{1\le i\le n} P_\theta(R_i, Z_i) \\
&= \sum_{1\le i\le n} \log P_\theta(R_i, Z_i) \\
&= \sum_{1\le i\le n} \log \Pi_{1\le j\le k} (\lambda_j P(R_i \mid Z_i = j))^{1_{Z_i=j}} \\
&= \sum_{1\le i\le n} \sum_{1\le j\le k} 1_{Z_i=j}[\log \lambda_j - \log \ell_j + \log(q^{d(R_i,I_j)}(1-2q)^{L-d(R_i,I_j)})] \\
&= \sum_{1\le i\le n} \sum_{1\le j\le k} 1_{Z_i=j}[\log \lambda_j - \log \ell_j + d(R_i, I_j) \log \frac{q}{1-2q} + L\log(1-2q)]
\end{aligned}
$$

Thus, after taking expectation, we get $E_{q(z|x,\theta^t)}[\ell(x, Z, \theta^{t+1})]$ as desired.

D.2 Feasibility of MSA in our setting

Note that when we only have substitution noise and if all the R_i originates from the same genomic location, the problem of $min_x \sum d(x, R_i)$ can be readily solved by a majority vote. We expect similar results regarding indel noise. However, we need to pre-process with an alignment phase before the majority vote. We thus introduce Algorithm majority-consensus-star-alignment.

1 Compute alignment of R_1 and R_j where $j \ge 2$
2 for j = 2 to n, use the alignment of R_1 and R_j to form introduce gaps to previous alignment with the principle of "once a gap always a gap"
3 Take column-wise majority to form x^*
4 return x^*

We note that in the alignment, we use the scoring scheme of (1, -1 ,-1 ,-10) for match, insertion, deletion, substitution. It is because pure substitution noise is rare in current long read technology. We also note that when there is a run of alphabet, we will push the gap towards the end of the alignment. For example CCAAATT is aligned to CCAA_TT.

Theorem D.1 *Let $\{R_i\}_{1\le i\le n}$ be a set of string with alphabets in $\{A, C, G, T\}$ of length $\{\ell(R_i)\}_{1\le i\le n}$ where $\ell(R_i) > n > 5$. If $\forall i \ne j, d(R_i, R_j) = 2$ and $\exists x^*$ such that $\forall i, d(x^*, R_i) = 1$ then the majority-consensus-star-alignment can find the optimizer of $\min_x \sum d(x, R_i)$.*

Proof We can break it down into the following three steps. A high level intuition is that we are randomly placing an error on R_i generated from the same source, so, a simple majority vote should just work after doing an initial alignment.

1. Note that x^* is the optimizer. If we define $R_{n+1} = R_1$, we have, $\forall x, \sum_{1\le i\le n} d(x, R_i) = \frac{1}{2}\sum_{1\le i\le n}[d(x, R_i) + d(x, R_{i+1})] \ge \frac{1}{2}\sum_{1\le i\le n} d(R_i, R_{i+1}) = n$ But since $\sum_{1\le i\le n} d(x^*, R_i) = \sum_{1\le i\le n} 1 = n$, we know that x^* is the optimizer.
2. Second, we assume we input the ground truth x^* as a read, we will find that the algorithm give x^* as the output.
   The reason is as follows. Let e_i be the edit introduced by R_i when aligned to $x*$. Note that $e_i \ne e_j$ if $i \ne j$ otherwise, $d(R_i, R_j) = 0$. So, it means that e_i cannot win the majority vote at the end because $n \ge 6$ and $|\{A, C, G, T, -\}| = 5$, so entry at $x*$ will be voted instead.
3. Finally, we find that the alignment with x^* is the same as that without it as input.
   The reason is as follows. We have the notation of $M(A, B)$ as the alignment of A and B when x^* is the first input, and $M_S(A, B)$ as the alignment of A and B when x^* is the input. We claim that a small lemma, which says that $\forall j, M(R_1, R_j) = M_S(R_1, R_j)$. Note that it suffices because no gaps are introduced without conflicting some R_i. Then with the lemma, we have alignment of every reads be identical with and without x^*, and by step 1 and 2, we know that the algorithm will output the right optimizer. Now we proceed to show the lemma. First note e_i corresponds to edit on $x*$ for R_i. Recall that, e_i has to be distinct due to $d(R_i, R_j) = 2$. Now consider , without loss of generality, e_1, e_2 and their corresponding location when x^* is the input. We define runs of alphabets that e_i lands on under M_S as r_i. Now, we exhaust the cases on r_i.
   (a) There exists at least one other run between r_1 and r_2. For example, AAAA-CCCTTT vs AAA-CCCTT- Since putting e_1, e_2 on M_S gives two edits between r_1, r_2, we cannot shift the alphabets at the middle to give the same edit distance. This means that the same alignment shows up under M so as to conserve the same edit distance. Moreover, as the - is always put to the end of run, we will have that consistent under both M_S and M too.
   (b) r_1, r_2 are neighboring runs. For example, CCC-TTT vs CCCCTT-. Shifting of run at r_1, r_2 will cause substitution error, so it is not used under M_S. Thus, r_1, r_2 will have the same alignment too under M to conserve the edit distance of 2.
   (c) r_1, r_2 are on the same run. For example, CCCC– vs CCCCC while x^*gives CCCC- Since the - is always put at the end of the run, we have the alignment conserved under M and M_S.

□

We note that, in our implementation of BIGMAC, we use ClustalW2[16] to do the core of multiple sequence alignment. We first use MUMmer to get a rough anchors of the reads and then we chop up the reads into smaller Kmers. Then, we group the related Kmers together use ClustalW2 to do the multiple sequence alignment.

D.3 An interesting repeat

There is an interesting case which can justify why we need the EM algorithm for some tough cases. Consider the situation in Fig 5. The correct matching is the one that follows row by row. However, there exists matching at the interior such that the polymorphic sites are still consistent (as shown in the figure). Moreover, if we only consider abundance information alone, this repeat cannot be resolved as well (in the sense that we cannot find the correct matching). However, if we consider both the abundances and the polymorphism together during the decision making, we can identify the correct linkage. That is why we introduce the parameter formulation to incorporate both of these quantities.

Figure 5: An example regarding why it requires abundances and edit distance should be considered together

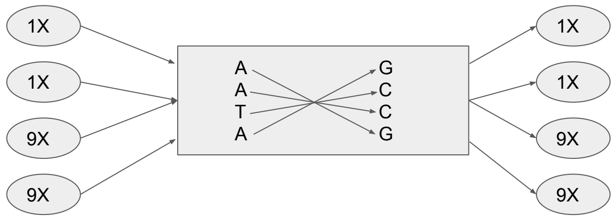

Appendix E: Commands for datasets

Commands for using BIGMAC on synthetic data and real data are all based on the following commands.

```
$ python -m srcRefactor.misassemblyFixerLib.mFixer destF mPath
$ python -m srcRefactor.repeatPhaserLib.aSplitter destF mPath
```

FinisherSC, SSPACE_LongRead and PBJelly are run at their default settings.
In particular, the commands used to run them are as follows.

```
FinisherSC :
$ python finisherSC.py dest mPath

PBJelly :
$ Jelly.py setup Protocol.xml
$ Jelly.py mapping Protocol.xml
$ Jelly.py support Protocol.xml
$ Jelly.py extraction Protocol.xml
$ Jelly.py assembly Protocol.xml
$ Jelly.py output Protocol.xml

SSPACE\_LongRead :
$  perl SSPACE-LongRead.pl -t 20 -c LC.fasta -p LR.fasta -b e2e/
```

The protocol.xml has the following setting for BLASR, `<blasr>-minMatch 8 -minPctIdentity 70 -bestn 1 -nCandidates 20 -maxScore -500 -nproc 20 -noSplitSubreads</blasr>`

Moreover, we note that you can reproduce results regarding BIGMAC by running `python reproduce.py` to download data, dependencies and run the tools. The results is saved in allinone.txt

Appendix F: Detailed Quast reports

The Quast reports for various comparison for synthetic data and dataset 1,2,3 are in the following tables.

Appendix G: Future work

It would be interesting to apply similar ideas to hybrid data. Moreover, it would also be interesting to investigate the optimal way to use abundance information.

Table 6: Synthetic data (Comparison with Breaker only and HGAP results). All statistics are based on contigs of size ≥ 500 bp, unless otherwise noted (e.g., "# contigs (≥ 0 bp)" and "Total length (≥ 0 bp)" include all contigs).

Assembly	Original	Breaker only	BIGMAC end-to-end
# contigs (≥ 0 bp)	**2**	4	**2**
# contigs (≥ 1000 bp)	**2**	4	**2**
Total length (≥ 0 bp)	**10000000**	**10000000**	9999992
Total length (≥ 1000 bp)	**10000000**	**10000000**	9999992
# contigs	**2**	4	**2**
Largest contig	**5000000**	2512000	4999998
Total length	**10000000**	**10000000**	9999992
Reference length	10000000	10000000	10000000
GC (%)	50.01	50.01	50.01
Reference GC (%)	50.01	50.01	50.01
N50	**5000000**	2512000	4999998
NG50	**5000000**	2512000	4999994
N75	**5000000**	2488000	4999994
NG75	**5000000**	2488000	4999994
L50	**1**	2	**1**
LG50	**1**	2	2
L75	**2**	3	**2**
LG75	**2**	3	**2**
# misassemblies	2	**0**	**0**
# misassembled contigs	2	**0**	**0**
Misassembled contigs length	10000000	**0**	**0**
# local misassemblies	0	0	0
# unaligned contigs	0 + 0 part	0 + 0 part	0 + 0 part
Unaligned length	0	0	0
Genome fraction (%)	100.000	100.000	100.000
Duplication ratio	1.000	1.000	1.000
# N's per 100 kbp	0.00	0.00	0.00
# mismatches per 100 kbp	**0.00**	**0.00**	0.05
# indels per 100 kbp	**0.00**	**0.00**	2.38
Largest alignment	2512000	2512000	**4999998**
NA50	2512000	2512000	**4999998**
NGA50	2512000	2512000	**4999994**
NA75	2488000	2488000	**4999994**
NGA75	2488000	2488000	**4999994**
LA50	2	2	**1**
LGA50	2	2	2
LA75	3	3	**2**
LGA75	3	3	**2**

Table 7: Dataset 1 (Comparison with Breaker only and HGAP results): All statistics are based on contigs of size ≥ 500 bp, unless otherwise noted (e.g., "# contigs (≥ 0 bp)" and "Total length (≥ 0 bp)" include all contigs).

Assembly	Original	Breaker only	BIGMAC end-to-end
# contigs (≥ 0 bp)	**130**	199	131
# contigs (≥ 1000 bp)	130	197	**129**
Total length (≥ 0 bp)	**30499818**	29452892	29273543
Total length (≥ 1000 bp)	**30499818**	29452752	29273403
# contigs	130	197	**129**
Largest contig	**8887616**	8615553	8615553
Total length	**30499818**	29452752	29273403
Reference length	30128987	30128987	30128987
GC (%)	56.54	57.45	57.68
Reference GC (%)	56.98	56.98	56.98
N50	818655	758280	**4352719**
NG50	1595590	567256	**4352719**
N75	**274801**	157172	**274801**
NG75	**277114**	132279	256020
L50	4	4	**3**
LG50	**3**	5	**3**
L75	23	28	**14**
LG75	22	32	**16**
# misassemblies	18	**4**	7
# misassembled contigs	15	**4**	7
Misassembled contigs length	16357196	**536534**	1785642
# local misassemblies	**6**	**6**	9
# unaligned contigs	0 + 0 part	0 + 0 part	0 + 0 part
Unaligned length	0	0	0
Genome fraction (%)	**98.189**	96.217	96.325
Duplication ratio	1.033	1.016	**1.010**
# N's per 100 kbp	0.00	0.00	0.00
# mismatches per 100 kbp	33.76	**22.38**	44.80
# indels per 100 kbp	7.13	**5.40**	63.44
Largest alignment	**8631596**	8615553	8615553
NA50	758280	758280	**4351628**
NGA50	758280	567256	**4351628**
NA75	227835	148337	**262515**
NGA75	**254545**	132279	181075
LA50	5	4	**3**
LGA50	5	5	**3**
LA75	26	29	**14**
LGA75	25	32	**17**

Table 8: Dataset 2 (Comparison with Breaker only and HGAP results). All statistics are based on contigs of size $\geq$ 500 bp, unless otherwise noted (e.g., "# contigs ($\geq$ 0 bp)" and "Total length ($\geq$ 0 bp)" include all contigs).

Assembly	Original	Breaker only	BIGMAC end-to-end
# contigs ($\geq$ 0 bp)	477	382	**351**
# contigs ($\geq$ 1000 bp)	477	371	**341**
Total length ($\geq$ 0 bp)	**32897488**	29572416	29605579
Total length ($\geq$ 1000 bp)	**32897488**	29569477	29603092
# contigs	477	374	**344**
Largest contig	4673711	4673711	4673711
Total length	**32897488**	29571716	29605331
Reference length	66662626	66662626	66662626
GC (%)	47.38	48.81	48.80
Reference GC (%)	46.01	46.01	46.01
N50	**397611**	354308	**397611**
N75	38471	59190	**75666**
L50	**9**	13	12
L75	101	70	**57**
# misassemblies	187	**25**	28
# misassembled contigs	176	**21**	22
Misassembled contigs length	18192123	**8079336**	8582043
# local misassemblies	22	**18**	19
# unaligned contigs	39 + 7 part	30 + 7 part	**29 + 8 part**
Unaligned length	1646412	**982915**	993710
Genome fraction (%)	41.946	41.941	**41.995**
Duplication ratio	1.118	1.023	**1.022**
# N's per 100 kbp	0.00	0.00	0.00
# mismatches per 100 kbp	**1.58**	1.97	8.68
# indels per 100 kbp	**8.63**	9.02	37.15
Largest alignment	4547258	4547258	4547258
NA50	**369454**	333580	**369454**
NA75	32926	47209	**56711**
LA50	**12**	15	14
LA75	123	81	**68**

Table 9: Dataset 3 (Comparison with Breaker only and HGAP results). All statistics are based on contigs of size $\geq$ 500 bp, unless otherwise noted (e.g., "# contigs ($\geq$ 0 bp)" and "Total length ($\geq$ 0 bp)" include all contigs).

Assembly	Original	Breaker only	BIGMAC end-to-end
# contigs ($\geq$ 0 bp)	185	154	**145**
# contigs ($\geq$ 1000 bp)	185	149	**140**
Total length ($\geq$ 0 bp)	**17393660**	13844743	13912664
Total length ($\geq$ 1000 bp)	**17393660**	13843875	13911796
# contigs	185	149	**140**
Largest contig	3968563	3968563	3968563
Total length	**17393660**	13843875	13911796
Reference length	7883268	7883268	7883268
GC (%)	61.18	60.96	60.98
Reference GC (%)	61.71	61.71	61.71
N50	257044	**359704**	**359704**
NG50	3968563	3968563	3968563
N75	82370	82649	**99878**
NG75	**3924590**	474671	517104
L50	**5**	7	7
LG50	1	1	1
L75	38	29	**27**
LG75	**2**	5	5
# misassemblies	26	**11**	14
# misassembled contigs	20	**5**	**5**
Misassembled contigs length	5470082	**4234268**	4328506
# local misassemblies	2	2	2
# unaligned contigs	118 + 0 part	121 + 1 part	**115 + 2 part**
Unaligned length	5585886	**5543409**	5553281
Genome fraction (%)	**99.983**	99.982	99.982
Duplication ratio	1.498	**1.053**	1.060
# N's per 100 kbp	0.00	0.00	0.00
# mismatches per 100 kbp	**0.18**	0.24	2.30
# indels per 100 kbp	**8.70**	22.88	23.60
Largest alignment	**3924590**	1719755	1719755
NA50	137772	**284436**	**284436**
NGA50	**1719755**	569978	576251
NGA75	**1452284**	474671	517104
LA50	11	**10**	**10**
LGA50	**2**	4	4
LGA75	**3**	7	7

Table 10: Dataset 1 (Comparison with other tools) : All statistics are based on contigs of size ≥ 500 bp, unless otherwise noted (e.g., "# contigs (≥ 0 bp)" and "Total length (≥ 0 bp)" include all contigs).

Assembly	original	BIGMAC	finisherSC_e2e	jelly_e2e	SSPACE_e2e
# contigs (≥ 0 bp)	130	131	**53**	100	86
# contigs (≥ 1000 bp)	130	129	**53**	100	86
Total length (≥ 0 bp)	30499818	29273543	29883342	**30619263**	30589751
Total length (≥ 1000 bp)	30499818	29273403	29883342	**30619263**	30589751
# contigs	130	129	**53**	100	86
Largest contig	8887616	8615553	8887616	**8889022**	8887616
Total length	30499818	29273403	29883342	**30619263**	30589751
Reference length	30128987	30128987	30128987	30128987	30128987
GC (%)	56.54	57.68	57.14	56.54	56.54
Reference GC (%)	56.98	56.98	56.98	56.98	56.98
N50	818655	4352719	2531294	4642330	**4657611**
NG50	1595590	4352719	2531294	4642330	**4657611**
N75	274801	274801	415024	418480	**493683**
NG75	277114	256020	399053	**818655**	**818655**
L50	4	**3**	**3**	**3**	**3**
LG50	3	3	3	3	3
L75	23	14	12	**6**	**6**
LG75	22	16	13	**5**	**5**
# misassemblies	18	**7**	32	19	32
# misassembled contigs	15	**7**	23	16	20
Misassembled contigs length	16357196	**1785642**	20096169	21804531	17545253
# local misassemblies	**6**	9	11	9	36
# unaligned contigs	**0 + 0 part**	**0 + 0 part**	**0 + 0 part**	0 + 11 part	**0 + 0 part**
Unaligned length	**0**	**0**	**0**	33217	**0**
Genome fraction (%)	98.189	96.325	98.330	**98.423**	98.189
Duplication ratio	1.033	**1.010**	1.030	1.034	1.037
# N's per 100 kbp	**0.00**	**0.00**	**0.00**	**0.00**	294.00
# mismatches per 100 kbp	**33.76**	44.80	73.10	34.06	33.96
# indels per 100 kbp	7.13	63.44	23.53	9.39	**6.69**
Largest alignment	8631596	8615553	8631596	**8631646**	8631596
NA50	758280	**4351628**	2530093	3871007	3854031
NGA50	758280	**4351628**	1537643	3871007	3854031
NA75	227835	262515	304665	**361412**	361362
NGA75	254545	181075	304665	**414429**	**414429**
LA50	5	**3**	**3**	**3**	**3**
LGA50	5	**3**	4	**3**	**3**
LA75	26	14	16	**8**	**8**
LGA75	25	17	16	**7**	**7**

Table 11: Dataset 2 (Comparison with other tools) : All statistics are based on contigs of size ≥ 500 bp, unless otherwise noted (e.g., "# contigs (≥ 0 bp)" and "Total length (≥ 0 bp)" include all contigs).

Assembly	original	BIGMAC	finisherSC_e2e	jelly_e2e	SSPACE_e2e
# contigs (≥ 0 bp)	477	351	447	403	**307**
# contigs (≥ 1000 bp)	477	341	447	403	**307**
Total length (≥ 0 bp)	32897488	29605579	32870423	**34484366**	33520228
Total length (≥ 1000 bp)	32897488	29603092	32870423	**34484366**	33520228
# contigs	477	344	447	403	**307**
Largest contig	4673711	4673711	4673711	4673711	4673711
Total length	32897488	29605331	32870423	**34484366**	33520228
Reference length	66662626	66662626	66662626	66662626	66662626
GC (%)	47.38	48.80	47.40	46.90	47.38
Reference GC (%)	46.01	46.01	46.01	46.01	46.01
N50	397611	397611	654163	**1585584**	1568442
NG50	-	-	-	17013	14909
N75	38471	75666	43018	61775	**95133**
L50	9	12	8	**6**	7
LG50	-	-	-	329	294
L75	101	57	89	65	**45**
# misassemblies	187	**28**	192	271	255
# misassembled contigs	176	**22**	168	246	165
Misassembled contigs length	18192123	**8582043**	18393113	24250973	23415983
# local misassemblies	22	**19**	22	37	101
# unaligned contigs	39 + 7 part	29 + 8 part	34 + 7 part	38 + 23 part	**17 + 5 part**
Unaligned length	1646412	**993710**	1594170	1760782	1479235
Genome fraction (%)	41.946	41.995	41.999	**43.521**	41.946
Duplication ratio	1.118	**1.022**	1.117	1.128	1.146
# N's per 100 kbp	**0.00**	**0.00**	**0.00**	**0.00**	1857.80
# mismatches per 100 kbp	**1.58**	8.68	4.39	15.40	**1.58**
# indels per 100 kbp	8.63	37.15	16.06	71.69	**8.49**
Largest alignment	4547258	4547258	4547258	4547258	4547258
NA50	369454	369454	401563	**742006**	737193
NA75	32926	**56711**	33995	46245	42004
LA50	12	14	11	**9**	**9**
LA75	123	**68**	113	90	82

Table 12: Dataset 3 (Comparison with other tools) : All statistics are based on contigs of size ≥ 500 bp, unless otherwise noted (e.g., "# contigs (≥ 0 bp)" and "Total length (≥ 0 bp)" include all contigs).

Assembly	original	BIGMAC	finisherSC_e2e	jelly_e2e	SSPACE_e2e
# contigs (≥ 0 bp)	185	145	162	133	**97**
# contigs (≥ 1000 bp)	185	140	162	133	**97**
Total length (≥ 0 bp)	17393660	13912664	17391031	**18003698**	17738519
Total length (≥ 1000 bp)	17393660	13911796	17391031	**18003698**	17738519
# contigs	185	140	162	133	**97**
Largest contig	3968563	3968563	3968563	3971059	**4319145**
Total length	17393660	13911796	17391031	**18003698**	17738519
Reference length	7883268	7883268	7883268	7883268	7883268
GC (%)	61.18	60.98	61.19	61.19	61.18
Reference GC (%)	61.71	61.71	61.71	61.71	61.71
N50	257044	359704	996532	1103847	**1266912**
NG50	3968563	3968563	3968563	3971059	**4319145**
N75	82370	99878	97964	128718	**290104**
NG75	3924590	517104	3924590	3927083	**3985906**
L50	5	7	**3**	**3**	**3**
LG50	1	1	1	1	1
L75	38	27	27	19	**10**
LG75	**2**	5	**2**	**2**	**2**
# misassemblies	26	**14**	25	27	43
# misassembled contigs	20	**5**	17	21	23
Misassembled contigs length	5470082	**4328506**	5465644	9434182	10736561
# local misassemblies	**2**	**2**	**2**	**2**	5
# unaligned contigs	118 + 0 part	115 + 2 part	99 + 0 part	66 + 14 part	**50 + 0 part**
Unaligned length	5585886	**5553281**	5602837	6149028	5791170
Genome fraction (%)	**99.983**	99.982	**99.983**	**99.983**	**99.983**
Duplication ratio	1.498	**1.060**	1.496	1.504	1.516
# N's per 100 kbp	**0.00**	**0.00**	**0.00**	**0.00**	1944.13
# mismatches per 100 kbp	0.18	2.30	**0.16**	0.60	0.18
# indels per 100 kbp	8.70	23.60	**5.14**	6.39	8.70
Largest alignment	3924590	1719755	3924590	**3925633**	3924590
NA50	137772	**284436**	152488	107893	126445
NGA50	**1719755**	576251	**1719755**	**1719755**	**1719755**
NGA75	1452284	517104	1452284	**1453076**	1452284
LA50	11	**10**	**10**	13	12
LGA50	**2**	4	**2**	**2**	**2**
LGA75	**3**	7	**3**	**3**	**3**
